# Identification of Recombinant Aichivirus D in Cattle, Italy

**DOI:** 10.3390/ani14223315

**Published:** 2024-11-18

**Authors:** Francesco Pellegrini, Gianvito Lanave, Francesca Caringella, Georgia Diakoudi, Anna Salvaggiulo, Alessandra Cavalli, Alessandro Papaleo, Barbara Di Martino, Michele Camero, Krisztián Bányai, Jelle Matthijnssens, Vito Martella

**Affiliations:** 1 Department of Veterinary Medicine, Università Aldo Moro di Bari, 70010 Valenzano, Italy; francesco.pellegrini@uniba.it (F.P.); gianvito.lanave@uniba.it (G.L.); francescacaringella91@gmail.com (F.C.); georgia.diakoudi@uniba.it (G.D.); anna.salvaggiulo@uniba.it (A.S.); alessandra.cavalli@uniba.it (A.C.); alessandro.papaleo@uniba.it (A.P.); michele.camero@uniba.it (M.C.); 2 Department of Veterinary Medicine, Università degli Studi di Teramo, 64100 Teramo, Italy; bdimartino@unite.it; 3 Department of Pharmacology and Toxicology, University of Veterinary Medicine, 1078 Budapest, Hungary; bkrota@hotmail.com; 4 Szentágothai Research Centre, University of Pécs, 7624 Pécs, Hungary; 5 Department of Laboratory Medicine, Medical School, University of Pécs, 7624 Pécs, Hungary; 6 Department of Microbiology, Immunology and Transplantation, Rega Institute, KU Leuven, 3000 Leuven, Belgium; jelle.matthijnssens@kuleuven.be

**Keywords:** Kobuvirus, cattle, recombination, metagenomics, Aichivirus

## Abstract

Kobuviruses (KoVs) are a group of small, non-enveloped RNA viruses classified in the genus *Kobuvirus* within the *Picornaviridae* family, comprising Aichivirus species A to F. KoVs have been identified in humans and several mammals, including domestic ungulates. This study investigated the presence of KoVs in a collection of bovine stool samples (*n* = 38) obtained from animals with enteritis or without clinical signs. By RT-PCR screening, KoV RNA was detected in 10/38 animals (26.3%). Six of the ten positive animals had enteric signs. On sequence analysis of the amplicons, eight strains were related to species Aichivirus B, commonly identified in cattle. In contrast, two strains (ITA/2019/572-1 and ITA/2020/bovine/30-2), displayed the highest nt identity (up to 97.1%) to cattle, yak, and goat Aichivirus D strains. On whole genome analysis, strains ITA/2019/572-1 and ITA/2020/30-2 showed 88.9% nt identity to each other and 87.8–90.3% nt to the bovine kobuvirus strain CHN/2021/ON730709 identified in China. Interestingly these three Aichivirus D strains showed a recombinant makeup, clustering with D1 genotype in the capsid region and with D2 genotype in the non-structural genes. These findings suggest that Aichivirus D KoVs are common components of livestock virome. Understanding the genetic diversity of KoVs in animals will be useful to improve the diagnostics and gather epidemiological data.

## Simple Summary

Kobuviruses (KoVs) possess a wide host range and have been associated with enteric disease in several host species. On screening of 38 stool samples collected from healthy (*n* = 21) and diarrheic (*n* = 17) animals, using a pan-KoV RT-PCR, viral RNA was detected in 10/38 (26.3%) samples. Six strains (including two Aichivirus D strains) were from animals with enteric signs. Applying whole genome sequencing, the Aichivirus D strains (ITA/2019/572-1 and ITA/2020/30-2) showed a close relatedness with a Chinese Aichivirus D strain and a possible recombinant nature. Understanding the genetic diversity of KoVs in animals will be useful in improving diagnostics and filling epidemiological gaps.

## 1. Introduction

Viral enteric infections may profoundly impact the health and productivity of cattle herds, chiefly affecting young calves, and often leading to fatal consequences [1]. The viruses frequently associated with bovine enteric disease are rotavirus A, coronavirus, bovine viral diarrhea virus, and astroviruses [2,3]. However, other viruses have been detected as common components of the bovine enteric virome, including kobuviruses (KoVs) [4].

KoVs are a group of small, non-enveloped RNA viruses classified in the genus *Kobuvirus* within the family *Picornaviridae* [5]. They possess a single-stranded, positive-sense RNA genome approximately 8.2–8.4 kilobases in length. The viral genome is enclosed in a capsid comprising 60 identical subunits. The RNA of KoVs contains 5′ and 3′ untranslated regions (UTRs) with a single large open reading frame (ORF) encoding for a polyprotein of 2436–2437 amino acids (aa) that undergoes protease processing to yield a leader protein (L), three structural viral proteins (VP0, VP3, and VP1) and seven non-structural proteins (NSPs) (2A-2C and 3A-3D). The VP1 capsid protein is the most variable structure and mainly elicits an immune response [6].

KoVs were first discovered in 1989 from an outbreak of gastroenteritis in human patients in Aichi Prefecture, Japan [7]. Subsequently, similar viruses have been reported in a multitude of animal hosts. Based on the genetic diversity of the complete polyprotein, VP0-VP3-VP1, 2C, and 3CD regions, KoVs are currently classified into six species, formerly known as Aichivirus (AiV) A to F, and 20 genetic types indicated with numbers [8].

Kobuvirus aichi, known as AiV A, has been found in several host species including humans (A1) [9], dogs (A2) [10], rodents (A3, A6–A10) [11], domestic cats (A4) [12], and birds (A5) [13]. Kobuvirus bejaponia, known as AiV B, has been found mostly in cattle (B1) [14], but it is also present in mustelids (B2) [15] and sheep (B3) [16]. Kobuvirus cebes, known as AiV C, is associated with goats (C2) [17] and pigs (C1) [18], while Kobuvirus dekago, or AiV D, has recently been discovered in Japanese black cattle [19]. Kobuvirus femyomini, or AiV F, has been identified in chiropterans (types F1 and F2) [20] and Kobuvirus ecuni, or AiV E, in rabbits (E1) [21].

Several studies have investigated the role of KoVs in enteric diseases of cattle. Bovine KoV (strain U-1) was first identified as a laboratory cell culture contaminant in 2003 in Japan [14]. Bovine KoVs have been subsequently reported in studies worldwide in animals with and without enteric signs in different age groups [22]. Epidemiological information from studies in several countries has been summarized in a review on bovine kobuviruses [22]. Overall, the incidence of kobuvirus in animals with enteric signs is reported to be 5.3–66.6%, whilst the incidence of kobuvirus in asymptomatic animals is 4.9–25.0%. On sequence analysis, the various KoV strains in cattle have shown genetic diversification, with several lineages, yet are all classified in the unique species AiV B1 [22]. In 2016, KoVs distantly related to AiV B1 were identified in Japan [19]. Based on the genome sequence, the prototype KoV strains (JPN/2014/Kago-1-22 and JPN/2015/Kago-2-24) have been classified into a novel species (AiV D) and proposed as two different genotypes, D1 and D2, respectively. Using specific primers, similar KoVs have been detected, respectively, in 16.9% and 10.4% of animals with enteritis from the same geographical area, but not in animals from other prefectures [8]. AiV D viruses have been subsequently reported in studies in China in yak (*Bos grunneris*) [23] and small ruminants [24]. In this study, we report the identification and characterization of AiV D in cattle in Italy.

## 2. Materials and Methods

### 2.1. Samples Collection

A convenience sampling collection was used in the study. Sample collection covered a period of 3 months, spanning from October 2019 to January 2020. A total of 6 farms were sampled in Taranto (5, Apulia region) and Cosenza (1, Calabria region), Italy, ranging in size from 50 to 500 head of cattle. In detail, 38 stool samples were collected either from animals showing enteric signs (*n* = 17) (acute diarrhea, weight loss, anorexia) or from healthy calves (*n* = 21). The age ranged from 20 days to 96 months old (Supplementary Table S1). The number of samples per farm varied from 2 to 16, sampling a least one healthy animal for each with enteritis.

### 2.2. Sample Preparation and Nucleic Acid Extraction

For sample preparation, the NetoVir protocol was used [25]. Briefly, stool samples were aliquoted and diluted with sterile PBS to create a 10% suspension. The homogenization was performed by Qiagen TissueLyser (Qiagen^TM^, Hilden, Germany) with a frequency of 25/s, followed by centrifugation at 16,000× *g* for 3 min. The extraction of nucleic acids was performed using the Indispin Pathogen DNA/RNA Mini Kit (Indical^®^, Leipzig, Germany) from 400 μL of the supernatants, according to the manufacturer’s instructions. Finally, nucleic acid was eluted (100 μL) and stored at −80 °C until later use.

### 2.3. RT-PCR Screening for Kobuvirus

A pan-KoV set of primers UNIV-kobu-F/UNIV-kobu-R was used for reverse transcription-polymerase chain reaction (RT-PCR). The primers are designed to amplify a 217-bp region of the viral RNA-dependent RNA polymerase complex (RdRp) of all known KoV species [26]. The amplicons were run on a 1.5% agarose gel containing a fluorescent dye (GelRed^®^ Nucleic Acid Gel Stain; Biotium, Fremont, CA, USA) at 90 V for 50 min and visualized on a Gel Doc imaging system (Bio-Rad Laboratories, Hercules, CA, USA). Specific amplicons of about 200 nt in length were visualized and excised from gel for purification (Invitrogen™, PureLink™ Quick Gel Extraction Kit) and sent for Sanger sequencing (Eurofins Genomics, Ebersberg, Germany).

### 2.4. Random Amplification and Illumina Sequencing

A modified version of the Whole Transcriptome Amplification (WTA) protocol (Sigma-Aldrich) was performed [27]. After a first step of denaturation at 95 °C for 2 min, the RNA was reverse transcribed using primers with a semi-degenerate 3′ end and a universal 5′ end (universal primers). The cDNA was amplified with a random PCR amplification for 17 cycles. The WTA PCR product was purified using the MSB^®^ Spin PCRapace kit (Invitek Molecular, Berlin, Germany). WTA amplification product was quantified using Qubit dsDNA HS assay kit (Thermo Fisher Scientific, Waltham, MA, USA). Library preparation was performed using an adjusted protocol of the Nextera XT Library Preparation Kit (Illumina, San Diego, CA, USA). The size of the library was checked with an A2100 Bioanalyzer (Agilent Technologies, Santa Clara, CA, USA) with a High Sensitivity DNA chip, to evaluate the length distribution of the obtained fragments. The samples were sequenced on an Illumina NextSeq 500 platform (2 × 150 bp paired end).

### 2.5. Whole Genome Sequencing of Kobuviruses

The complete genome sequence of KoVs was reconstructed using a primer walking strategy with multiple sets of primers designed in conserved regions. The 5′ and 3′ ends sequences were obtained by Rapid Amplification of complementary DNA End (RACE) protocols [28,29] using SuperScript-III Taq kit (Invitrogen™, Life Technologies, Milan, Italy) for reverse transcription LaTakara PCR kit (TaKaRa Bio Europe S.A.S, France) for amplification. The PCR products were run on 1.5% agarose gel and the bands of the expected size were excised and purified (PureLink™ Quick Gel Extraction Kit, Invitrogen™, Life Technologies, Milan, Italy) and sequenced with Sanger technology.

### 2.6. Sequence and Phylogenetic Analysis

Samples that tested positive by the pan-KoV RT-PCR were sequenced and analyzed to identify homologous hits in the NCBI database using the Basic Local Alignment Search Tool (BLAST) http://www.ncbi.nlm.nih.gov (accessed on 9 March 2024) with default parameters. Data obtained by Illumina sequencing were analyzed in parallel by the software package Geneious Prime version 2024.2 (Dotmatics, Germany) and the online software Genome Detective Virus Tool v 2.48 (GDVT) [30].

The KoV sequences were aligned with cognate KoV strains retrieved from the GenBank database (NCBI, accessed on 15 March 2024) using MAFFT v7.490 [31]. The best substitution model parameters for phylogenetic inference and assessment of selection pressure were predicted using “Find the best protein DNA/Protein Models” implemented in MEGA X version 11.0.13 [32]. Bootstrap replication was set to 1000 to assess the reliability of the inferred tree.

The software tools SimPlot v1.3.0 [33], RdP v5.58 [34], and Python package Recan v0.5 [35] with default parameters were used to assess potential recombination events in the genome sequences.

### 2.7. GenBank Sequence Submission

The nucleotide sequences of strains ITA/2019/572-1, ITA/2020/30-1 and ITA/2020/30-2 employed for phylogeny were deposited in the GenBank database under accession numbers PQ360972, PQ360973 and PQ360974 respectively.

### 2.8. Statistical Analysis

A Fisher’s exact test was used to find possible association between the presence of KoV and enteric signs in the 38 animals. Statistical calculations were performed using GraphPad Prism v8.1.2 program Intuitive Software for Science, San Diego, CA, USA. The statistical significance level was always set at 0.05. 

## 3. Results

### 3.1. Pan-Kobuvirus RT-PCR Screening

On pan-KoV RT-PCR screening, 10 samples out of 38 (26.3%) tested positive. The samples were collected from three different meat farms located in Taranto (Apulia, Italy) and from a farm situated in Cosenza (Calabria, Italy). Of the 10 KoV-positive samples, 4 were from healthy animals, and 6 were from juveniles showing enteric signs.

**Figure 1:**
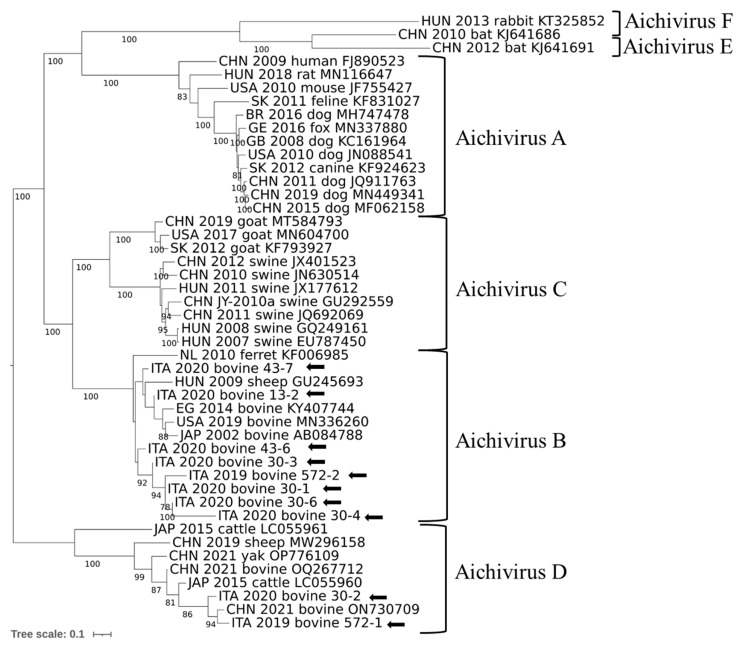
Phylogenetic reconstruction based on partial RdRp sequences obtained in this study (arrows) and reference sequences obtained from Genbank. Statistical support was determined using 1000 bootstrap replicates, with gamma distribution and invariant sites.

Interrogation of NCBI databases using the BLASTn tool (NCBI; accessed in March 2024) confirmed the specificity of the amplicons (nucleotide [nt] identity 94.2–97.7%). Identity among the 10 sequences obtained in this study ranged from 90.6% to 99.4%. A phylogenetic tree was generated based on the partial RdRp sequence (Figure 1). In the tree, most sequences were grouped with AiV B1 genotype strains, whilst two sequences were grouped with AiV D2 genotype.

### 3.2. Full Genome Sequence Analysis and Amplification

The WTA protocol (Sigma-Aldrich) was used to generate libraries from the 38 samples and sequenced on an Illumina NextSeq 500 platform. Overall, sequencing yielded 0.40–3.85 GB of data for the 38 samples (mean = 2.52 GB). The number of reads obtained ranged between 589 and 849,166 (mean = 72,244.60, Median = 8782.12; St. Dev. = 164,041.21), with an average length of 50–134 bp. The FASTq data were analyzed using Genome Detective Virus Tool v 2.48 (GDVT). The sequencing reads were classified in various taxa, including CRESS DNA virus, parvovirus, porprismacovirus, rotavirus, picobirnavirus, torovirus, enterovirus, kobuvirus, parapoxvirus, and mastadenovirus. Overall, five samples (572/19–1, 572/19–2, 30/20–1, 30/20–2, and 30/20–4) contained reads classified at genus level as KoV, ranging from 189 to 43,063 reads (mean = 13,716.66; median = 1032.5).

**Figure 2:**
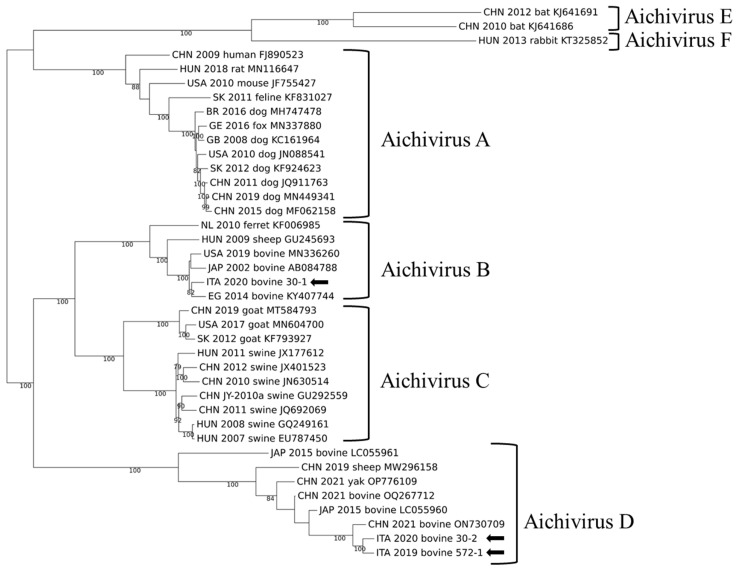
Phylogenetic reconstruction based on complete KoV genomes obtained in this study (arrows) and hit sequences obtained from GenBank. Statistical support was determined using 1000 bootstrap replicates, with gamma distribution and invariant sites.

For three strains, genome coverage after Illumina sequencing was above 90% (572/19-1, 30/20-2, and 30/20-1), with a depth of coverage ranging between 30.7 and 688.0 × (mean = 425.66). The complete genome sequence of strains ITA/2019/572-1, ITA/2020/30-2, and ITA/2019/572-1 were obtained. In the complete genome-based phylogenetic analysis, strain ITA/2020/30-1 was characterized as AiV B1, while strains ITA/2019/572-1 and ITA/2020/30-2 were related to AiV D strains identified in cattle, yak, and sheep in Asia [19,23,24] (Figure 2).

**Figure 3:**
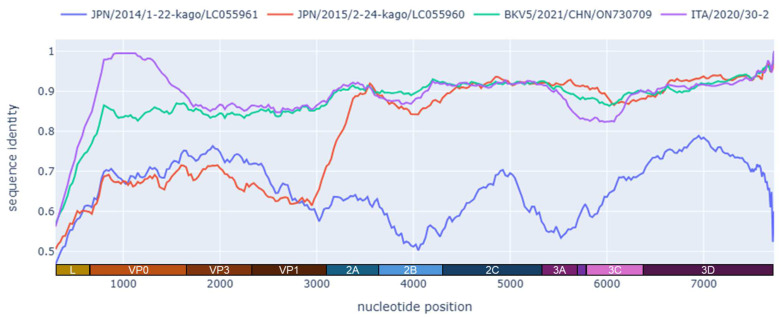
Whole genome identity plot of strain ITA/2019/572-1 in comparison with strains ITA/2020/30-2, JAP/2015/2-24-kago/LC055960 (D2), JAP/2014/1-22-kago/LC055961 (D1), and BKV5/2021/CHN/ON730709. The plot was generated using the Python package Recan v0.5 with a window of 200 nt and a shift of 50 nt.

On pairwise sequence comparison, strains ITA/2019/572-1 and ITA/2020/30-2 displayed the highest genetic relatedness (87.8–90.3% nt identity) to a bovine KoV complete genome sequence (CHN/2021/BKV5, GenBank accession no. ON730709) detected in feces from cattle in China in 2021 (unpublished) (Figure 3), whilst nt identities to other AiV D strains were 77.5–80.7%. Strain ITA/2020/30-2 was correlated to other bovine KoVs (AiV B1), showing the highest nucleotide identity (91.6%) to a complete genome detected in the USA (IL35164, GenBank accession no. MN336260).

The genome coding sequences of strains ITA/2019/572-1 and ITA/2020/30-2, excluding the terminal UTR regions, were respectively 7509-nt and 7518-nt in length, with a single ORF encoding a predicted polyprotein of 2503 and 2506 aa, respectively. The genome layout of the polyprotein was similar to that of other members of the genus Kobuvirus comprising an L protein, three structural proteins (VP0, VP3, and VP1), and seven NSPs (2A to 2 C and 3A to 3D). The cleavage sites, predicted by sequence alignments and NCBI Conserved Domain Search, https://www.ncbi.nlm.nih.gov/Structure/cdd/wrpsb.cgi (accessed on 2 September 2024), were Q/G (L/VP0), Q/H (VP0/VP3), Q/P (VP3/VP1), Q/C (VP1/2A), Q/G (2A/2B), QS (2B/2C), QG (2C/3A), QA (3A/3B), QG (3B/3C), and Q/S (3C/3D).

When comparing the aa sequences, the ICTV species demarcation criteria were considered [8]. In the 2C+3CD regions, the aa identity of ITA/2019/572-1 and ITA/2020/30-2 with AiV D2 reference strain (JPN/2015/Kago-2-24, LC055960) was 96.3% and 96.4%, respectively. However, in the P1 region (VP0, VP3, and VP1), the aa identity for strain JPN/2015/Kago-2-24 (LC055960, AiV D2) and JPN/2014/Kago 1-22 (LC055961, AiV D1) was significantly lower (61.3–66.1% and 62.4–67.9%, respectively), exceeding the 30% divergence threshold (Table 1).

**Figure 4:**
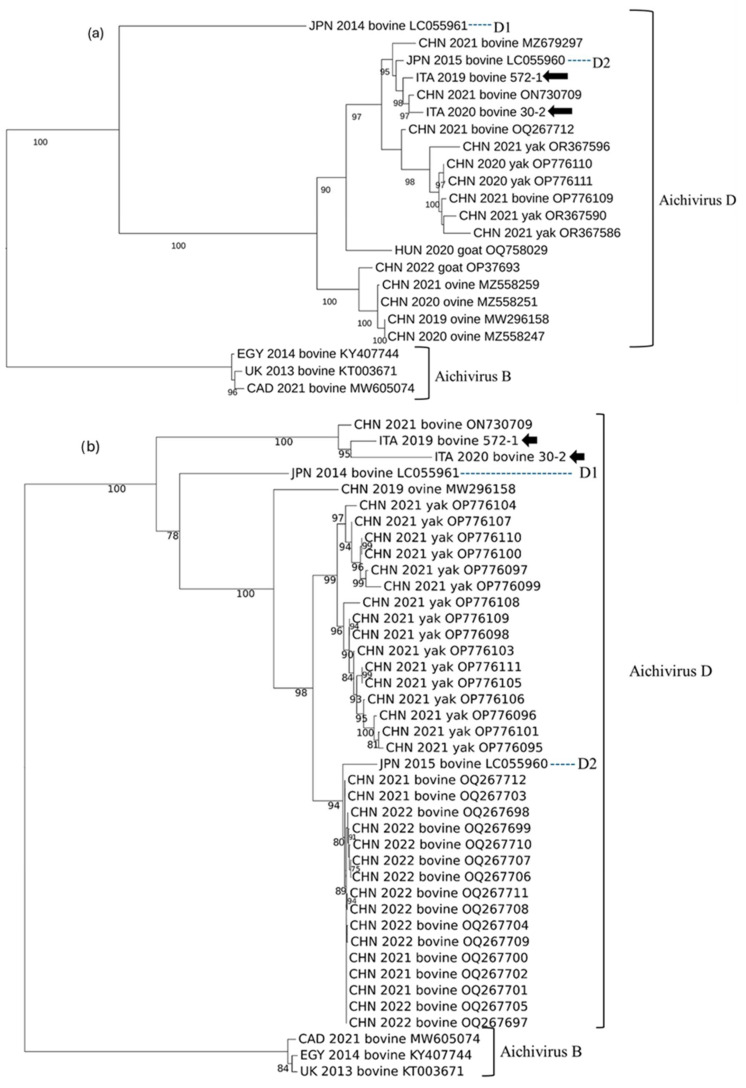
Phylogenetic reconstruction based on the NSPs (2A to 3D) (**a**) and P1 (VP0, VP3, VP1) (**b**) KoV sequences obtained in this study (arrows) and hit sequences obtained from GenBank. Statistical support was determined using 1000 bootstrap replicates, with gamma distribution and invariant sites.

Trees based on the P1 and the seven NSPs (2A to 3D) were also generated (Figure 4). In the P1-based tree, the strains ITA/2019/572-1, ITA/2020/30-2, and CHN/2021/BKV5 (ON730709) segregated together, in a branch rooted with the D1 strain JPN/2014/Kago 1-22 (LC055961). In the tree based on the NSPs, the strains ITA/2019/572-1, ITA/2020/30-2, and CHN/2021/BKV5 (ON730709) segregated together with the D2 strain JPN/2015/Kago-2-24 (LC055960) (AiV D2).

Recombination analysis identified a possible recombination event affecting the P1 region, seemingly located near the junction of the VP1 and A2 genes (Figure 3).

### 3.3. Evaluation of Statistical Association

In the 38 animals, no significant association was found between RT-PCR positivity to KoV and the presence of enteric signs (*p* = 0.29).

## 4. Discussion

The epidemiology of KoVs has been the subject of studies in several animal species in recent years. Their possible association with enteric disease has been hypothesized in various animal hosts, including humans, domestic carnivores, and pigs [12,36,37]. In cattle, KoVs have been involved in multifactorial enteritis outbreaks in co-infection with other enteric pathogens [38,39,40] and have been detected in both healthy and sick animals [41,42]. A possible role of KoVs as enteric pathogens in cattle has been hypothesized in several studies, although this has not been demonstrated firmly [40,41,43,44]. However, experimental infection with a genotype D2 KoV strain isolated on VERO cells produced diarrhea and depression in 2-month-old yak calves. The clinical signs started 3 days after infection and peaked at 6 days. Some of the infected animals were euthanized on day 8 post-infection and gross pathological lesions were observed in the duodenum and jejunum, while the virus was also detected in most internal organs, indicating systemic infection [23].

In this study, using a pan-KoV RT-PCR, an overall positivity rate of 26.3% (10/38) was observed among the animals sampled from six farms. KoV RNA was detected in five out of six farms. Six of the ten KoV-positive animals exhibited enteric signs. Since we screened a small number of animals after a convenience sampling, the epidemiological value of our findings is limited. Most (80%, 8/10) bovine KoV strains detected in our study were related to bovine AiV B1, a species/genotype commonly reported in cattle [42,43]. However, two strains, ITA/2019/572-1 and ITA/2020/30-2, were more related genetically to AiV D strains detected in ruminants in Asia [23]. The highest aa identity was to the bovine strain CHN/2021/BKV5 (ON730709) and it was 93.0–94.4% in the polyprotein, 85.7–90.9% in the P1, and 96.4–96.3% in the 2C+3CD. The sequences were also compared to the prototype Japanese D1 and D2 strains. In the 2C+3CD region, there was a high identity (95.2–96.7% aa) to the genotype D2 strain JPN/2015/LC055960 and a lower identity (67.9–68.1% aa) to the D1 strain JPN/2014/LC055961. In the functional P1 region (VP0-VP3-VP1), there was a low aa identity to both AiV D1 (62.4–67.9%) and D2 (61.3–66.1%), below the threshold established for species classification. Based on the ICTV criteria, KoVs of the same species display a divergence of <30%aa in the polyprotein, <30% aa in the P1, and <20% aa in the 2C+3CD. Accordingly, the strains ITA/2019/572-1, ITA/2020/30-2, and CHN/2021/BKV5 would not meet, strictly, the criteria for classification into the AiV D species, if considering only the P1 gene, challenging the ICTV classification criteria. By observing the identity plot (Figure 3), it was clear that from the 2A encoding gene onwards (regions P2/2A-2B-2C and P3/3A-3B-3C-3D), there was a high degree of conservation among the AiV D strains, while upstream, in the P1 region, a high genetic diversity was observed. Interestingly, in the P1-based tree, the strains ITA/2019/572-1, ITA/2020/30-2, and CHN/2021/BKV5 (ON730709) segregated together, in a branch rooted with the D1 strain JPN/2014/Kago 1-22 (LC055961), while in the tree based on the NSPs (i.e., P2 and P3 functional regions), the three strains segregated together with the D2 strain JPN/2015/Kago-2-24 (LC055960). These inconsistencies in the phylogenetic patterns could suggest a recombination event between a D2 KoV strain, and an, as of yet, uncharacterized KoV strain, distantly related to JPN/2014/Kago 1-22 (LC055961) (Figure 3).

Genetic recombination is a driving force in the evolution of single-stranded RNA viruses [45,46]. Picornaviruses are characterized by marked genetic variability, often due to intraspecies/intertypic recombination events, and, less frequently, recombination at the interspecies level [47]. These events seem to increase the adaptability and pathogenicity of viral strains [48,49,50,51]. In many picornavirus genera, recombination has been demonstrated [52,53,54], with the cross-over points being frequently located at the 3′ end of the P1 region that encodes for the structural proteins [55]. Recombination within the P1 region has been rarely reported, usually between viruses of the same serotype [56,57]. Regardless, the Italian strains ITA/2019/572-1, ITA/2020/30-2, and the Chinese strain CHN/2021/BKV5 (ON730709) retained a conserved genomic composition, based on our analyses, thus indicating that these viruses are circulating in cattle in distinct geographical areas. Likewise, recombination patterns and mosaic genome organization have been observed in human AiVs (Kobuvirus Aichi) [58,59]. The possibility of recombination events between human and animal picornaviruses has not been demonstrated, although interspecies transmission seems to have occurred on more occasions. Swine vesicular disease virus (SVDV) emerged around the 1960s worldwide and originated from a Coxsackievirus B5, a common human enterovirus [60]. In 1975, however, an SVDV (strain T75), likely originated from a human coxsackievirus B4, caused an epizootic in pigs in the Soviet Union, demonstrating that these events are not uncommon [61]. Cross-species transmission has been reported for kobuviruses in cattle, goats, and pigs, highlighting the complexity of the evolutionary pathways of kobuviruses in animals [51,62]. However, animal-like KoVs have not been identified in humans, thus far.

Based on our data and literature, bovine KoVs are genetically diverse, and this could challenge the attempts to understand their pathogenic role since different KoV strains could possess different virulence phenotypes [23]. Interestingly, the two AiV D strains identified in this study were detected in calves with enteritis. Experimental infections with B1 and D1 KoV types could help assess if all bovine KoVs possess this virulent phenotype. However, further studies are needed to assess the viability of bovine KoVs detected in stools. Likewise, epidemiological investigations using diagnostic tools with type-specific resolution could be helpful to investigate the pathogenetic role, in any, of bovine KoVs.

## 5. Conclusions

In this study, a high (26.3%) KoV prevalence was found in calves. Sequence analysis revealed a marked genetic diversity and a potential novel KoV type of recombinant origin. Gathering information on the genetic diversity of KoVs will help design more reliable diagnostic tools and improve our knowledge of these enteric viruses.

## Figures and Tables

**Table 1:** Identity of strains CHN/2021/ON730709, ITA/2019/572-1 and ITA/2020/30-2 2C, 3CD, P1, and Polyprotein AA sequences with strains JPN/2014/LC055961 and JPN/2015/LC055960, considered as reference strains for AiV D1 and D2, respectively.

	2C+3CD (aa) (>79% Identity) (<20% Divergence)			P1 (aa) (>69% Identity) (<30% Divergence)			Polyprotein (aa) (>69% Identity) (<30% Divrgence)		
	ON730709	LC055960 (D2)	LC055961 (D1)	ON730709	LC055960 (D2)	LC055961 (D1)	ON730709	LC055960 (D2)	LC055961 (D1)
ON730709	x	96.4	68.0	x	67.1	68.9	x	85.1	63.6
ITA/2020/30-2	96.4	95.2	68.1	85.7	61.3	62.4	93.0	82.3	61.1
ITA/2019/572-1	96.3	96.7	67.9	90.9	66.1	67.9	94.4	85.2	61.0
D1		68.3	x		73.5	x		65.3	x
D2		x	68.3		x	73.5		x	65.3

## Data Availability

The original contributions presented in the study are included in the article and Supplementary Material, further inquiries can be directed to the corresponding author.

## Supplementary Materials

The following supporting information can be downloaded at: https://www.mdpi.com/article/10.3390/ani14223315/s1, Table S1: Data, health conditions, and results of the animals sampled, from October 2019 to January 2020.
