# Supplementary material for: Identification of Recombinant Aichivirus D in Cattle, Italy
Source: Animals (Basel). 2024 Nov 18;14(22):3315. doi: 10.3390/ani14223315 (PMC11591108; doi:10.3390/ani14223315)
Supplement: Supplementary file 1 [file animals-14-03315-s001.zip › animals-3253273-supplementary.pdf]

Table S1. Data, health conditions, and results of the animals sampled, from October 2019 to January 2020.

PROTOCOL	DATE	SITE	AGE	SEX	PRODUCTION	HEALTH STATUS	GENERAL CONDITION	STOOL	Kobuvirus NGS	Kobuvirus RT-PCR
572/19 - 1	24/10/2019	TARANTO 1°	30 DAYS	F	MEAT	SICK	DEPRESSED	DIARRHEA WITH BLOOD	+	+
572/19 - 2	24/10/2019	TARANTO 1°	20 DAYS	M	MEAT	SICK	DEPRESSED	DIARRHEA WITH BLOOD	+	+
572/19 - 3	24/10/2019	TARANTO 1°	20 DAYS	M	MEAT	HEALTHY	NORMAL	NORMAL		
572/19 - 4	24/10/2019	TARANTO 1°	25 DAYS	F	MEAT	HEALTHY	NORMAL	DIARRHEA		
572/19 - 5	24/10/2019	TARANTO 1°	20 DAYS	M	MEAT	SICK	DEPRESSED	DIARRHEA WITH BLOOD		
572/19 - 6	24/10/2019	TARANTO 1°	30 DAYS	M	MEAT	HEALTHY	NORMAL	SOFT		
13/20 - 1	14/01/2020	TARANTO 2°	48 MONTHS	F	MILK	HEALTHY	NORMAL	NORMAL		
13/20 - 2	14/01/2020	TARANTO 2°	30 DAYS	M	/	SICK	ANOREXIA	DIARRHEA (YELLOW)		+
30/20 - 1	17/01/2020	TARANTO 3°	20 MONTHS	M	MEAT	HEALTHY	NORMAL	NORMAL	+	+
30/20 - 2	13/01/2020	TARANTO 4°	20 MONTHS	M	MEAT	SICK	DEPRESSED	DIARRHEA	+	+
30/20 - 3	17/01/2020	TARANTO 3°	20 MONTHS	M	MEAT	HEALTHY	NORMAL	NORMAL		+
30/20 - 4	17/01/2020	TARANTO 3°	20 MONTHS	F	MEAT	SICK	ANOREXIA, DEPRESSED	DIARRHEA (YELLOW)	+	+
30/20 - 5	07/01/2020	TARANTO 3°	20 MONTHS	M	MEAT	HEALTHY	NORMAL	NORMAL		
30/20 - 6	17/01/2020	TARANTO 3°	20 MONTHS	M	MEAT	SICK	DEPRESSED	DIARRHEA (BLOOD)		+
30/20 - 7	07/01/2020	TARANTO 3°	36 MONTHS	F	MEAT	SICK	NORMAL	DIARRHEA		
30/20 - 8	17/01/2020	TARANTO 4°	20 MONTHS	M	MEAT	HEALTHY	NORMAL	NORMAL		
30/20 - 9	17/01/2020	TARANTO 3°	60 MONTHS	M	MEAT	HEALTHY	NORMAL	NORMAL		
30/20 - 10	17/01/2020	TARANTO 3°	48 MONTHS	M	MEAT	HEALTHY	NORMAL	NORMAL		
30/20 - 11	17/01/2020	TARANTO 3°	48 MONTHS	M	MEAT	SICK	DEPRESSED	DIARRHEA		
30/20 - 12	05/10/2019	TARANTO 3°	60 MONTHS	M	MEAT	SICK	DEPRESSED	DIARRHEA		
30/20 - 13	07/11/2019	TARANTO 5°	36 MONTHS	M	MEAT	HEALTHY	NORMAL	NORMAL		
30/20 - 14	07/11/2019	TARANTO 5°	96 MONTHS	M	MEAT	SICK	/	DIARRHEA (BLOOD AND MUCUS)		
43/20 - 1	22/01/2020	COSENZA 6°	36 MONTHS	M	MEAT	HEALTHY	NORMAL	NORMAL		
43/20 - 2	22/01/2020	COSENZA 6°	36 MONTHS	M	MEAT	HEALTHY	NORMAL	NORMAL		
43/20 - 3	22/01/2020	COSENZA 6°	36 MONTHS	M	MEAT	HEALTHY	NORMAL	NORMAL		
43/20 - 4	22/01/2020	COSENZA 6°	36 MONTHS	M	MEAT	HEALTHY	NORMAL	NORMAL		

43/20 - 5	22/01/2020	COSENZA 6°	36 MONTHS	M	MEAT	HEALTHY	NORMAL	NORMAL
43/20 - 6	22/01/2020	COSENZA 6°	36 MONTHS	F	MEAT	HEALTHY	NORMAL	NORMAL
43/20 - 7	22/01/2020	COSENZA 6°	36 MONTHS	F	MEAT	HEALTHY	NORMAL	NORMAL
43/20 - 8	22/01/2020	COSENZA 6°	36 MONTHS	M	MEAT	HEALTHY	NORMAL	NORMAL
43/20 - 9	22/01/2020	COSENZA 6°	36 MONTHS	M	MEAT	HEALTHY	NORMAL	NORMAL
43/20 - 10	22/01/2020	COSENZA 6°	36 MONTHS	M	MEAT	HEALTHY	NORMAL	NORMAL
43/20 - 11	22/01/2020	COSENZA 6°	36 MONTHS	F	MEAT	SICK	/	DIARRHEA
43/20 - 12	22/01/2020	COSENZA 6°	36 MONTHS	M	MEAT	SICK	/	DIARRHEA
43/20 - 13	22/01/2020	COSENZA 6°	36 MONTHS	M	MEAT	SICK	/	DIARRHEA
43/20 - 14	22/01/2020	COSENZA 6°	36 MONTHS	M	MEAT	SICK	DEPRESSED	DIARRHEA
43/20 - 15	22/01/2020	COSENZA 6°	36 MONTHS	M	MEAT	SICK	DEPRESSED	DIARRHEA
43/20 - 16	22/01/2020	COSENZA 6°	36 MONTHS	M	MEAT	SICK	NORMAL	DIARRHEA
